# Duodenal ulcer bleeding from a branch of the middle colic artery: A case report

**DOI:** 10.1097/MD.0000000000035955

**Published:** 2023-11-03

**Authors:** Yutaka Shishido, Eisei Mitsuoka, Yuma Tanigawa, Hodaka Ooki, Seiji Shio, Shuichi Monzawa, Masayuki Ishii, Koji Fujimoto

**Affiliations:** a Department of Gastrointestinal Surgery, Shinko Hospital, Kobe, Hyogo, Japan; b Department of Diagnostic Radiology, Shinko Hospital, Kobe, Hyogo, Japan; c Department of Gastroenterology, Shinko Hospital, Kobe, Hyogo, Japan.

**Keywords:** case report, gastrointestinal hemorrhage, interventional radiology, peptic ulcer hemorrhage, transcatheter arterial embolization

## Abstract

**Rationale:**

Duodenal ulcer bleeding is a potentially life-threatening condition commonly caused by the erosion of the duodenal arteries.

**Patient concerns:**

A 55-year-old male was referred to our hospital with abdominal pain for the past 3 days. Contrast-enhanced computed tomography of the abdomen revealed wall thickening in the descending part of the duodenum and a cystic lesion (27 × 19 mm) contiguous with the duodenum, with an accumulation of fluid. An esophagogastroduodenoscopy showed the significantly stenotic duodenum, which prevented passage of the endoscope and evaluation of the main lesion. Based on these findings, duodenal ulcer perforation and concomitant abscess formation were suspected. Two days after admission, he had massive hematochezia with bloody drainage from the nasogastric tube.

**Diagnoses:**

Emergency angiography revealed duodenal ulcer bleeding from the gastroduodenal artery and the branch artery of the inferior pancreaticoduodenal artery and middle colic artery (MCA).

**Interventions:**

The patient was treated with transcatheter arterial embolization (TAE) of the gastroduodenal artery, the branch vessel of the inferior pancreaticoduodenal artery, and the main trunk of the MCA.

**Outcomes:**

Hemostasis was achieved with TAE. The patient recovered uneventfully and undergone a gastro-jejunal bypass surgery for the duodenal stenosis 2 weeks after TAE. He was discharged without any abnormal complaints on postoperative day 12.

**Lessons:**

We have experienced a rare case of duodenal ulcer bleeding from a branch of the MCA. In patients with refractory upper gastrointestinal bleeding, careful evaluation of bleeding sites is recommended considering unexpected culprit vessels.

## 1. Introduction

Upper gastrointestinal (GI) bleeding is a common and potentially life-threatening condition that refers to bleeding from the esophagus, stomach, or duodenum. Peptic ulcer disease is the most common cause of upper GI bleeding, and can be classified into gastric and duodenal ulcers.^[1]^ Duodenal ulcer bleeding (DUB) is caused by the erosion of the duodenal arteries, such as the gastroduodenal artery (GDA),^[2,3]^ and has been associated with higher mortality, surgical requirement, and readmission rates than gastric ulcer bleeding.^[4]^ Therefore, it is crucial for clinicians to understand the treatment strategies and management options for acute DUB. Although endoscopic therapy is the gold standard for treating upper GI bleeding, interventional radiology (IVR) or surgical management is considered for patients with hemodynamic instability or those refractory to endoscopic therapy.^[5]^ With the recent advancement in IVR, transcatheter arterial embolization (TAE) is widely accepted as an effective and safe procedure to control acute massive DUB.^[6]^ However, in high-risk and complicated settings, treatment decisions are sometimes challenging, and discussion about multidisciplinary treatments among surgeons, gastroenterologists, and interventional radiologists is required to manage upper GI bleeding.^[5]^ In this report, we present a case of massive DUB that was not indicated for surgery or endoscopic treatment and was refractory to IVR, which was finally treated by embolizing the duodenal arteries and middle colic artery (MCA).

## 2. Case presentation

**Figure 1.**
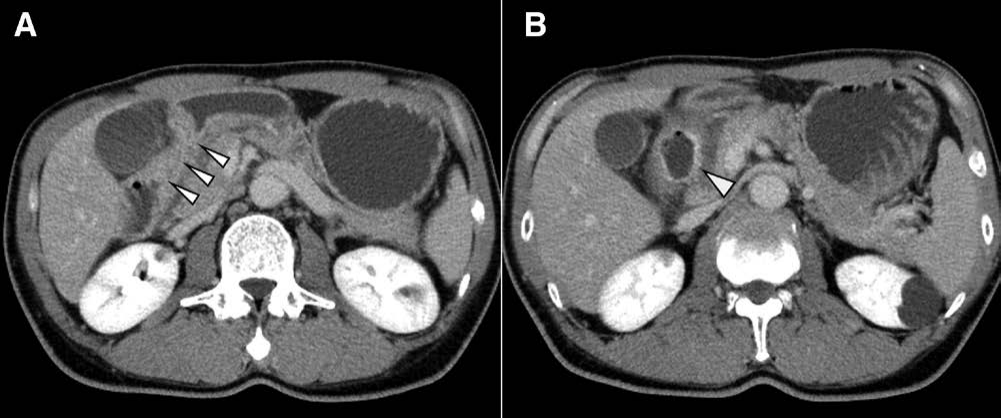
Contrast-enhanced computed tomography showed (A) wall thickening and luminal narrowing in the descending part of the duodenum (arrowheads) and (B) a cystic lesion approximately 27 × 19 mm contiguous to the duodenum (arrowhead).

A 55-year-old male was referred to our hospital for evaluation of abdominal pain for the past 3 days. He had a history of surgery for ruptured abdominal organs due to a traffic accident and for a duodenal ulcer more than 20 years earlier; however, the details were unavailable. He was not on any medication. Vital signs were within normal limits at the initial visit. Physical examination revealed tenderness in the right costal region without rebound or guarding. Blood examinations showed no abnormalities, with a hemoglobin (Hb) of 13.4 g/dL. Contrast-enhanced computed tomography (CECT) of the abdomen revealed wall thickening and luminal narrowing in the descending part of the duodenum (Fig. 1A). Furthermore, a cystic lesion approximately 27 × 19 mm was found, contiguous with the duodenum, with an accumulation of fluid (Fig. 1B). Based on these findings, duodenal ulcer perforation, and concomitant abscess formation were suspected. He was admitted to the hospital, where he was intubated with a nasogastric (NG) tube and administered proton pump inhibitors intravenously. Esophagogastroduodenoscopy was performed a day after he was admitted, revealing that the duodenal bulb was highly deformed with erosions and the superior part of the duodenum was significantly stenotic. Therefore, the endoscope could not pass through the duodenum, and the main lesion in the descending portion could not be evaluated. Two days after admission, he developed massive hematochezia with bloody drainage from the NG tube. His level of consciousness decreased, and his systolic blood pressure dropped to 60 to 70 mm Hg with a heart rate of 50 to 60 bpm. Blood tests showed a low Hb of 7.7 g/dL and a high blood urea nitrogen level of 26 mg/dL. CECT showed a mild high-density component in the duodenum, but no extravasation of contrast. A DUB was suspected, and emergency endoscopic therapy was planned. However, endoscopic hemostasis was considered unsuccessful by the gastroenterologists because the endoscope could not pass through the duodenum. After administration of extracellular fluid and 4 units of red blood cells (RBCs), the blood pressure and level of consciousness stabilized. Based on the CECT findings and absence of further increase in bloody drainage from the NG tube, it was assumed that there was no active bleeding and the patient was to be followed up without invasive treatment. However, about 12 hours later, the patient experienced massive blood stools again. Although 4 units of RBCs and 2 units of fresh frozen plasma (FFP) were administered, the patient became restless and went into hemorrhagic shock with rapidly increasing bloody drainage from the NG tube.

**Figure 2.**
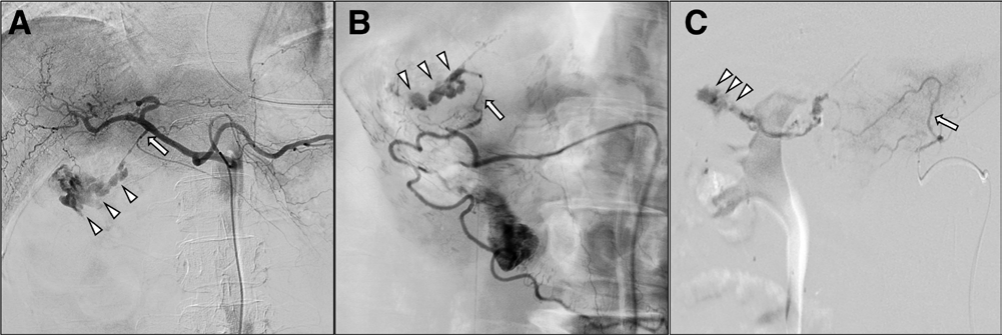
Angiography reveals (A) extravasation (arrowheads) from the gastroduodenal artery (arrow) and (B) extravasation (arrowheads) from the branch vessel of the inferior pancreaticoduodenal artery (arrow), (C) extravasation (arrowheads) from the branch vessel of the middle colic artery (arrow).

Emergency TAE was planned to achieve hemostasis. Angiography of the celiac artery revealed extravasation from the GDA and contrast agent migration into the duodenum (Fig. 2A). Furthermore, angiography of the superior mesenteric artery revealed extravasation from the branch artery of the inferior pancreaticoduodenal artery (IPDA) (Fig. 2B). Interventional radiologists embolized the main trunk of the GDA and branch vessel of the IPDA using N-butyl cyanoacrylate and ethyl ester of iodinated poppy seed oil fatty acid as embolic agents. Angiography of the celiac artery and superior mesenteric artery confirmed the disappearance of extravasation. He was admitted to the intensive care unit with a Hb of 3.9 g/dL. Multiple packs of RBC, FFP, and platelets were transfused to correct anemia and stabilize the patient. However, 2 hours after TAE, the patient went into hemorrhagic shock again with approximately 1000 mL of bloody drainage from the NG tube. CECT revealed extravasation from the duodenal wall, and repeated emergency TAE was planned. Although repeated celiac and superior mesenteric angiogram failed to reveal any bleeding, selective angiography of the MCA revealed extravasation from a branch vessel of the MCA (Fig. 2C). Due to the patient’s life-threating condition and urgent need for hemostasis, the main trunk of the MCA was embolized after discussing the possibility of colonic ischemia with GI surgeons and interventional radiologists. The cessation of extravasation was confirmed by angiography, and TAE was finally completed. The patient eventually received 42 units of RBCs, 12 units of FFP, and 25 units of platelets for this bleeding event.

**Figure 3.**
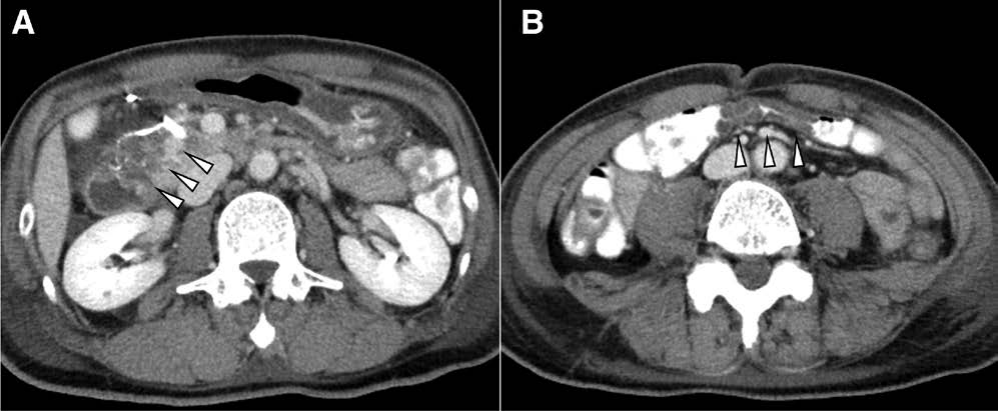
Contrast-enhanced computed tomography conducted 5 days after interventional radiology showed (A) diminished contrast effect in the pancreaticoduodenal region (arrowheads), and (B) partial edema and stenosis of the transverse colon (arrowheads).

Although the contrast effect in the pancreaticoduodenal region was diminished (Fig. 3A), CECT performed 5 days after TAE showed no evident extravasation. However, there was partial edema and stenosis of the transverse colon (Fig. 3B). The improvement of these abnormalities in the transverse colon was confirmed on CECT conducted 3 days later without any interventions. Two weeks after TAE, gastro-jejunal bypass surgery was performed because of severe stenosis of the duodenum. On laparotomy, the duodenum, and transverse colon were well-colored, and there were no obvious necrotic findings in the pancreaticoduodenal region. The patient recovered uneventfully and commenced oral feeding on postoperative day 4. The patient was discharged with an oral proton pump inhibitor on postoperative day 12. One month post operation, the patient was stable with no abnormal complaints, and esophagogastroduodenoscopy via the gastro-jejunal bypass tract revealed an ulcer scar in the duodenum without bleeding, mucosal necrosis, or pathological malignancy on biopsy.

## 3. Discussion

Upper GI bleeding is a common and potentially critical medical condition, with an incidence rate of 36 to 172 per 100,000 individuals.^[1]^ Despite recent medical advancements, mortality from upper GI bleeding remains relatively high, ranging from 3% to14%, which may be a consequence of shifting population demographics, with elderly patients having more medical comorbidities.^[1,7]^ Peptic ulcer disease is the most common cause of upper GI bleeding, accounting for 28% to 59% of all cases.^[1]^ In particular, DUB has a worse prognosis than any other non-variceal bleeding in terms of mortality, surgical requirement, and readmission to the hospital.^[4]^ One reason for the worse prognosis of DUB is that the GDA is located just behind the posterior duodenum, and erosion of this artery or its branches could cause massive bleeding.^[8]^ In addition to the GDA, arteries that feed the pancreaticoduodenal region, such as the IPDA and anterior superior pancreaticoduodenal artery, could also be culprit vessels for acute DUB.^[2,3]^ In this case, the duodenal ulcer and its perforation could have damaged the main stem of the GDA and branching arteries of the IPDA and MCA, causing severe upper GI bleeding. To our knowledge, there have been no reports of DUB from a branch of the MCA, which might be due to congenital anomaly or the development of collateral blood vessels associated with past surgical history for internal organ rupture and duodenal ulcer. Considering the rarity of the culprit vessels and their refractoriness to treatment, we believe it is worthwhile to report this case and discuss the treatment course.

In high-risk cases, treatment of DUB is challenging and requires a multidisciplinary approach involving various medical specialists, including surgeons, gastroenterologists, and interventional radiologists.^[5]^ Although endoscopic therapy is regarded as the gold standard treatment for upper GI bleeding, persistent or refractory bleeding occurs in approximately 10% of patients.^[9]^ For patients with an unsuccessful endoscopy, surgery or IVR is indicated as an alternative treatment. TAE is increasingly used in clinical practice because of its advancement and lower morbidity than surgery.^[10]^ However, a meta-analysis including 6 studies suggested that TAE could be associated with a higher rebleeding risk than surgery.^[11]^ Therefore, the treatment of choice for upper GI bleeding in high-risk patients remains controversial. In this case, endoscopic hemostasis was not achieved because the endoscope could not pass through the duodenum. Therefore, we only had 2 options for hemostasis: surgery and TAE. When the patient first developed symptoms of upper GI bleeding, he might have been able to tolerate the surgical intervention because his vital signs were quickly stabilized with volume resuscitation. However, the patient had a history of abdominal surgery, and a high degree of adhesion in the abdominal cavity was expected. Therefore, in the worst-case scenario, the patient might have had to undergo pancreatoduodenectomy to stop bleeding in the descending part of the duodenum, which would have been too invasive. When the patient went into hemorrhagic shock for the second time, TAE was the only option because resuscitation with blood transfusion could not be achieved. Moreover, in this case, not only the vessels feeding the pancreaticoduodenal region, but also a branch vessel of the MCA contributed to DUB, which was difficult to predict during surgery. Given the instability of the vital signs, invasiveness of surgery, and rare culprit vessels, we concluded that TAE was the appropriate treatment for this case.

The appropriate timing of initial therapy for DUB remains controversial. In terms of endoscopic therapy, endoscopy is generally recommended within 24 hours of the presentation of patients with upper GI bleeding.^[12]^ However, it is important to conduct early risk evaluation based on hemodynamic status and comorbidities and determine the appropriate treatment timing depending on the individual.^[5]^ Urgent endoscopy is recommended in patients with hemodynamic instability within 12 hours or even 6 hours after initial volume restitution.^[12,13]^ The Glasgow-Blatchford score (GBS), which is composed of clinical and laboratory data, was developed to predict a composite of clinical intervention or death.^[14]^ GBS ranges from 0 to 23, with higher scores indicating higher risk, and the 30-day mortality of patients with a score of 12 or higher was 16.1%.^[15]^ Based on the GBS, our case was considered high-risk with a score of 18 due to high blood urea nitrogen, low Hb, low systolic blood pressure, presence of melena, and syncope at the first manifestation of DUB. However, emergency TAE was not performed at the time for the following reasons. First, we considered active bleeding unlikely because of the lack of obvious extravasation on CECT and the absence of increased bloody drainage from the NG tube. However, although arterial-phase CECT has been reported to be accurate in detecting and localizing the bleeding site, it failed to detect GI bleeding in 7.7% of patients.^[16]^ Furthermore, discharge from the NG tube might not be informative in assessing upper GI bleeding.^[17]^ Hence, the probability of active bleeding cannot be ruled out. Second, given the lack of extravasation on CECT, we assumed that the bleeding site could not be identified on angiography, which could have resulted in an unsuccessful TAE. However, empirical embolization for massive DUB has been reported as a useful method for controlling bleeding without serious complications.^[18]^ Third, the patient developed bloody stool late at night on the weekend, and it was difficult to discuss the treatment plan with the medical team, including the interventional radiologists. However, given the risk in this patient and the possibility of active bleeding, TAE should have been performed as early as possible after the initial onset of DUB.

## 4. Conclusion

We have experienced a rare case of DUB treated with TAE of the duodenal arteries and a branch of the MCA. In patients with refractory DUB, careful evaluation of bleeding sites is recommended considering unexpected culprit vessels.

## Author contributions

**Conceptualization:** Yutaka Shishido.

**Investigation:** Yutaka Shishido.

**Resources:** Eisei Mitsuoka, Yuma Tanigawa, Hodaka Ooki, Seiji Shio, Shuichi Monzawa.

**Supervision:** Eisei Mitsuoka, Masayuki Ishii, Koji Fujimoto.

**Writing – original draft:** Yutaka Shishido.

**Writing – review & editing:** Yutaka Shishido, Eisei Mitsuoka, Shuichi Monzawa.
